# Transcriptome-Wide Analysis of Core Transcription Factors Associated with Defense Responses in Autotetraploid versus Diploid Rice under Saline Stress and Recovery

**DOI:** 10.3390/ijms242115982

**Published:** 2023-11-05

**Authors:** Yingkai Wang, Weilong Meng, Yan Ye, Xinfang Yu, Haiyuan Chen, Yuchen Liu, Minghong Xu, Ningning Wang, Fan Qi, Yujie Lan, Yan Xu, Jian Ma, Chunying Zhang

**Affiliations:** 1 Faculty of Agronomy, Jilin Agricultural University, Changchun 130000, China; wangyingkai99@163.com (Y.W.); mengweilong0212@163.com (W.M.); ye_yanyanyan@163.com (Y.Y.); qinghua1552911578@163.com (X.Y.); doctom42@163.com (H.C.); m13274645701@163.com (Y.L.); 13756579606@163.com (M.X.); wangnn826@163.com (N.W.); fan711998@163.com (F.Q.); lan814772@163.com (Y.L.); 15059008970@163.com (Y.X.); 2 Jilin Provincial Laboratory of Crop Germplasm Resources, Changchun 130000, China

**Keywords:** ABA-activated signaling, autotetraploid rice, circadian rhythm, recovery, saline stress, transcription factor

## Abstract

Saline stress is a major abiotic stress that inhibits plant growth and yields worldwide. The plant transcription factor (TF) family plays an important role in converting abiotic stress signals into gene expression changes. In this study, a transcriptome-based comparative analysis was performed to investigate the global gene expression of all the TFs in diploid and autotetraploid rice during the early stage of NaCl stress and recovery period. The phenotypic data indicated that the tetraploid rice exhibited a superior salt-tolerant ability compared to the diploid rice. A total of 55 TF families were co-expressed in the tetraploid and diploid rice, and the cumulative number of TF-expressed genes was relatively higher in the diploid rice than in the tetraploid rice at all time points. Unlike the diploid rice, the overall gene expression levels of the tetraploid rice were comparable to the control during recovery. The number of differentially expressed TFs (DE-TFs) in the tetraploid rice decreased after recovery, whereas it increased to a large extent in the diploid rice. GO and KEGG pathway enrichment analysis of the DE-TFs discovered the early switching of the ABA-activated signaling pathway and specific circadian rhythm in the tetraploid rice. Combining the PPI network and heatmap analysis, some core DE-TFs were found that may have potential roles to play in tetraploid salt tolerance. This study will pave the way for elucidating the complex network regulatory mechanisms of salt tolerance in tetraploid rice.

## 1. Introduction

Rice (*Oryza sativa* L.) is the primary source of energy for over half of the world’s population. Unfortunately, rice yields and the grain quality are deeply affected by unfavorable environmental factors, such as drought, salinity, or extreme temperatures [1,2]. High salinity is considered as one of the disastrous abiotic stresses that seriously impacts the growth, development, metabolism, productivity, and quality of rice plants [3]. During evolution, plants developed many sophisticated but effective strategies to cope with adverse environments, such as polyploidization and whole-genome duplication (WGD) [4]. Polyploidy is thought to provide a way out for sustained survival by enabling swift adaptation to rapidly changing environments [5]. In rice, tetraploid plants exhibit more resistance to saline stress than diploids [6,7]. However, the molecular basis underlying the increased stress resistance of polyploid rice remains unclear. Understanding the corresponding mechanisms of the rice response and tolerance to saline stress is a critical first step in developing innovative and rational strategies to improve the salt tolerance in rice.

Plants try to adapt to changing environmental factors through internal mechanisms to resist external pressures [2]. For instance, transcription factors (TFs) can interact with *cis* elements within the promoters and activate a range of stress-related genes to synthesize multiple functional proteins, enabling plants to survive. TFs play an important role in regulating gene transcription in a series of biological processes related to growth, development, and environmental stimuli [8,9]. Several TF families of rice, including NAC, MYB, bZIP, bHLH, WRKY, and AP2/ERF, promote stress adaptation by regulating the expressions of stress-responsive genes [10,11,12,13,14,15,16]. In addition, the dynamic changes of phytohormones have undeniable impacts on the normal growth and development of plants under saline stress [17]. Earlier studies showed that the indole-3-acetic acid (IAA)-related genes SAUR32, SAUR36, and ARF5, as well as the cell division-related gene IPT5, can improve salt tolerance by increasing the contents of IAA and cytokinin (CK) in the roots of apple rootstocks [18]. In the abscisic acid (ABA) signaling pathway, five genes related to ABA receptors in *Sophora alopecuroides* regulate stomatal closure by promoting ABA accumulation to alleviate the inhibition of photosynthesis by saline–alkali stress, enabling plants to better adapt to harsh environments [19]. Wang et al. reported that the salt tolerance in tetraploid rice was enhanced via lower sodium intake and associated with the epigenetic regulation of jasmonic acid (JA)-related genes [6]. As we know, each phytohormone has a recognized, unique signaling pathway [20]. Cross-regulation between these pathways occurs during signal transduction and transcriptional regulation processes. The cross-regulation of transcription can be achieved through TFs shared between pathways or the regulation of shared target genes by independent TFs [21]. Different TFs regulate genes in a dynamic manner at different times in response to stress. Just as the expressions of from tens to hundreds of TFs are regulated by ABA, JA, and ethylene (ET), it appears that all hormones act similarly [22,23,24,25]. Therefore, identifying the regulatory relationship from the perspective of transcription factors could be effective for accurately understanding transcriptional regulatory mechanisms.

High-throughput RNA sequencing (RNA-seq) has been recognized as one of the widespread tools for measuring gene transcriptional expression profiles, and it can provide a superior platform for a comprehensive understanding of the contribution of TFs to plant regulatory systems [26,27]. To date, studies on the transcriptional regulation of genes against saline stress in polyploid rice are limited. Although the importance of TFs in rice stress tolerance has been highlighted, more concern has been paid to expression analysis and the identification of single TF families based on high-throughput sequencing [28,29,30], resulting in a lack of overall understanding of transcriptional regulation. In the present research, we performed a transcriptome-based comparative analysis to investigate the global gene expression of all the TFs in salt-sensitive diploid and salt-tolerant autotetraploid rice during the early stage of NaCl stress and recovery period, and we established a regulatory network for the core DE-TFs, aiming to pave the way for elucidating the complex network regulatory mechanisms of salt tolerance in polyploid rice.

## 2. Results

### 2.1. Evaluation of Salt Tolerance of GFD–T and GFD–D

**Figure 1:**
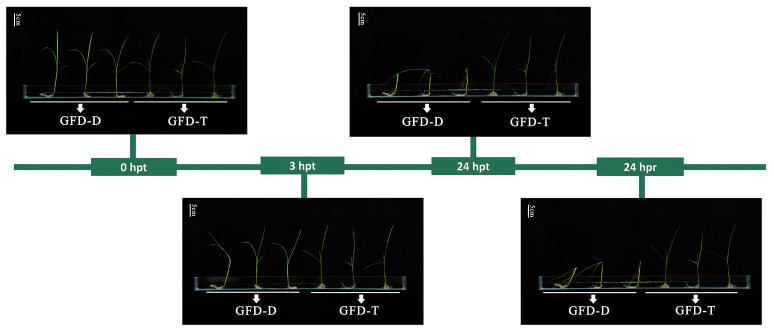
Phenotypic trend of enhanced salt tolerance in autotetraploid rice GFD–T. The trefoil-stage seedlings of GFD–T and GFD–D were treated with 125 mM NaCl for 0 h, 3 h, and 24 h, and the saline stress was removed for 24 h (recovery) (scale bar = 5 cm). D: diploid; T: tetraploid; hpt: hours post-treatment; hpr: hours post recovery. Experiments contained at least five independent biological replicates.

The differences between the GFD–T and GFD–D seedling responses to saline stress were evaluated via the phenotypic changes at different time points of stress reactivity and recovery. Previous studies have shown that rice is relatively tolerant to salt stress during the germination, tillering, and maturity stages but is sensitive during the seedling (50–120 mM NaCl) and reproductive (30–100 mM NaCl) stages [31,32,33]. Seedlings of GFD–D and GFD–T at the trefoil stage were cultured under a series of saline solutions (0, 50, 100, 125, 150, and 200 mM NaCl, Materials and Methods) for 24 h and then returned to nutrient solution for 24 h. We selected 125 mM NaCl as an optimal treatment for further study, as the seedlings were slightly affected at NaCl concentrations below 100 mM and exhibited severe necrosis at concentrations of 150 mM or higher. There were no significant differences in the growth or phenotypic characteristics between the GFD–T and GFD–D at 3 h post-treatment (hpt), but the GFD–D displayed severe leaf wilting and stem lodging at 24 hpt and 24 h post recovery (hpr), which suggested that the GFD–T had a better salt-tolerance ability compared with its diploid progenitor GFD–D (Figure 1).

### 2.2. Overview of TFs in Transcriptomes of GFD–T and GFD–D

**Figure 2:**
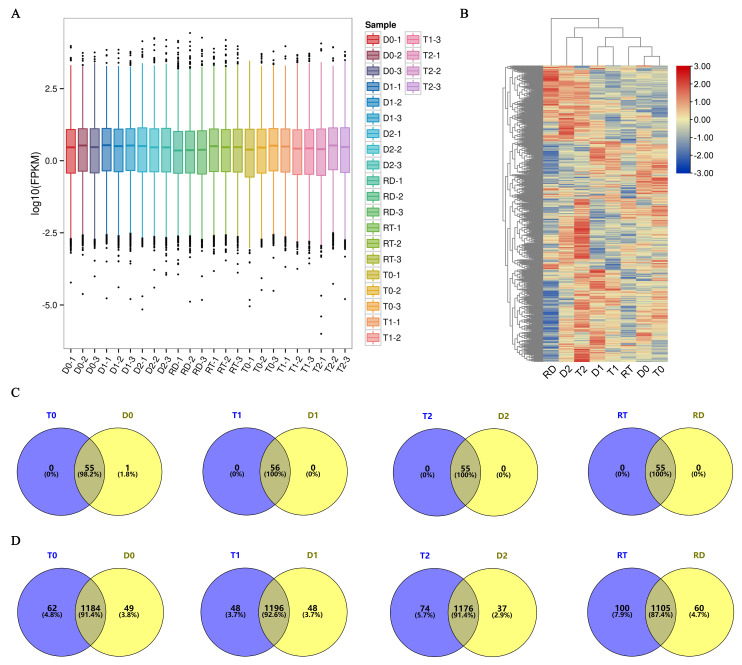
Genome-wide expression analysis of 56 TF families and their members in GFD–T and GFD–D during saline stress and recovery stage. (**A**) Boxplot of FPKM for gene expression levels. Abscissa symbolizes sample names and ordinate represents Log10 (FPKM). A boxplot for each region covers five statistics (from bottom to top: minimum, lower quartile, median, upper quartile, and maximum), and the outliers are shown in black dots. (**B**) Hierarchically clustered heatmap of global TF gene expression. Venn diagrams representing (**C**) common and specific TF families and (**D**) TF genes in GFD–T and GFD–D at all time points. D0/T0: diploid or tetraploid seedlings without NaCl stress; D1/T1: diploid or tetraploid seedlings treated with NaCl for 3 h; D2/T2: diploid or tetraploid seedlings treated with NaCl for 24 h; RD/RT: recovery culture of diploid or tetraploid seedlings for 24 h. TF: transcription factor.

To elucidate the TFs involved in the GFD–T salt tolerance during the early stage (first 24 h) of saline stress and recovery, a comparative transcriptomic analysis was carried out. We sequenced twenty-four cDNA libraries of GFD–T and GFD–D and obtained equivalent reads and coverage depths between replicates for each sample by comparing FPKM boxplots of the gene expression levels among all the genes under different experimental conditions (Figure 2A), which suggested that the RNA sequencing (RNA-seq) results were reliable. To further evaluate the reliability of the RNA-seq data, quantitative real-time polymerase chain reaction (qRT-PCR) was used to analyze the expression patterns with seven randomly selected genes. The results showed that the RNA-seq data were positively correlated (*R*^2^ = 0.815) with the qRT-PCR results, indicating the reliability of the RNA-seq data (Supplementary Figure S1 and Table S1). Furthermore, all the expressed TFs involved in the 56 TF families with count values > 30 were identified and clustered, which showed that the expressed TFs of the GFD–T and GFD–D aggregated in pairs at 0 hpt, 3 hpt, and 24 hpt, respectively. Notably, the TFs derived from the GFD–RT (recovery culture of tetraploids for 24 h) were grouped together with the TFs contained in the untreated samples, while the GFD–RD (recovery culture of diploids for 24 h)-derived TFs were grouped together with the 24 h post-treated samples (Figure 2B). At all time points, 1342 and 1347 unigenes from the 56 TF families were identified in the GFD–T and GFD–D, respectively (Supplementary Table S2). Among them, a total of 115 and 112 unigenes were expressed in the bHLH family and over 70 unigenes were expressed in the WRKY, ERF, MYB, bZIP, NAC, and C2H2 families in the GFD–T and GFD–D, respectively (Supplementary Table S2). And these seven family members comprised more than 21% of the total unigenes (Supplementary Table S2). Furthermore, 55 TF families (excluding LFY) co-expressed 1078 genes and 1126 genes at all time points in the GFD–T and GFD–D, respectively (Supplementary Figure S2A,B and Table S2). And one gene derived from the LFY family was specifically co-expressed in the GFD–T and GFD–D at 3 hpt (Figure 2C and Supplementary Table S2). Interestingly, the number of unigenes expressed at each time point in the GFD–T was generally higher than in the GFD–D, whereas the accumulated number of unigenes at all time points of the GFD–T was lower than that of the GFD–D, suggesting that the GFD–D activated more TF genes than the GFD–T in response to whole-process stress to save it from persecution (Figure 2D and Supplementary Table S2).

### 2.3. Intra-Group Comparison of Pairwise Differentially Expressed TFs and Gene Ontology Enrichment in GFD–T and GFD–D

**Figure 3:**
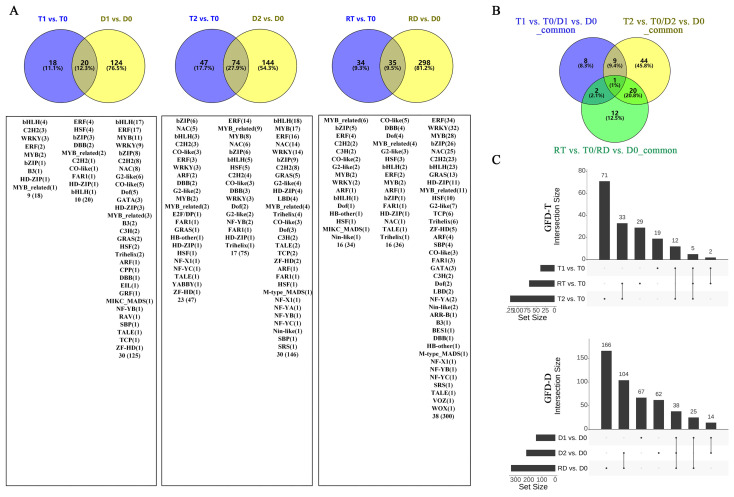

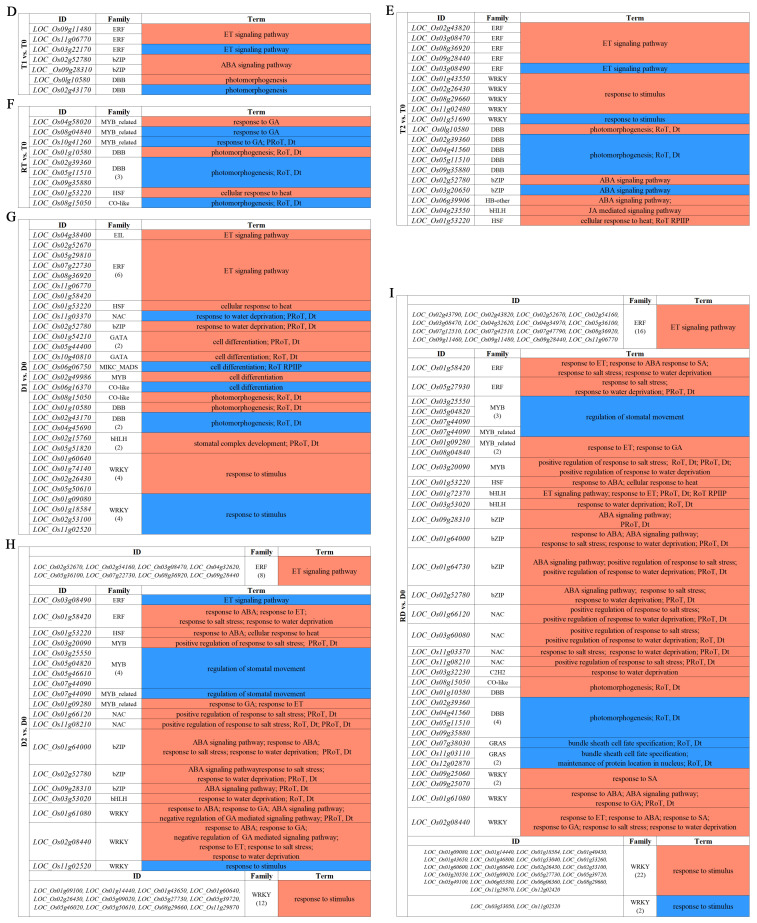
Comparison of pairwise DE-TFs and gene functional characteristics in GFD–T and GFD–D. Venn diagrams of (**A**) DE-TFs and (**B**) common DE-TFs at each time point of pairwise comparison group in GFD–T and GFD–D. (**C**) Upset plots derived unique and shared DE-TF genes at each time point of pairwise comparison group in GFD–T and GFD–D. (**D–I**) Gene Ontology enrichment of DE-TFs derived from intra-group comparisons in GFD–T and GFD–D. D0/T0: diploid or tetraploid seedlings without NaCl stress; D1/T1: diploid or tetraploid seedlings treated with NaCl for 3 h; D2/T2: diploid or tetraploid seedlings treated with NaCl for 24 h; RD/RT: recovery culture of diploid or tetraploid seedlings for 24 h. DE-TFs: differentially expressed transcription factors; ABA: abscisic acid-activated; ET: ethylene-activated; GA: gibberellin; JA: jasmonic acid; PRoT, Dt: positive regulation of transcription, DNA-templated; RoT RPⅡP: regulation of transcription from RNA polymerase II promoter; RoT, Dt: regulation of transcription, DNA-templated; SA: salicylic acid.

To identify the stress-response TFs differentially expressed at different time points of the tetraploid (T0, T1, T2, and RT) and diploid (D0, D1, D2, and RD) rice, we compared the gene expression values of the intra-group pairwise comparisons between the GFD–T (T1 vs. T0, T2 vs. T0, and RT vs. T0) and GFD–D (D1 vs. D0, D2 vs. D0, and RD vs. D0). The Venn diagrams showed that there were 18, 47, and 34 unique differentially expressed TFs (DE-TFs) from 9, 23, and 16 TF families in T1 vs. T0, T2 vs. T0, and RT vs. T0, respectively. In contrast, D1 vs. D0, D2 vs. D0, and RD vs. D0 contained 124, 144, and 298 unique DE-TFs from 30, 30, and 38 TF families, respectively (Figure 3A and Supplementary Table S3). These data displayed that the number of DE-TFs in the GFD–T increased with the duration of stress, and then decreased at RT, whereas in the GFD–D, the number of DE-TFs continued to increase. Notably, the number of DE-TFs in RT vs. T0 was quite low compared to RD vs. D0 (Figure 3A). Furthermore, there was one DE-TF gene (*LOC_Os02g32590* (HSF)) co-expressed among T1 vs. T0/D1 vs. D0_common, T2 vs. T0/D2 vs. D0_common, and RT vs. T0/RD vs. D0_common, suggesting that this gene may play a key role in both GFD–T and GFD–D in the response to saline stress (Figure 3B). The upset plots indicated that five DE-TFs were co–expressed among T1 vs. T0, T2 vs. T0, and RT vs. T0, and 38 DE-TFs were co–expressed among D1 vs. D0, D2 vs. D0, and RD vs. D0 (Figure 3C and Supplementary Table S4). Among them, two genes (*LOC_Os01g51690* (WRKY) and *LOC_Os01g10580* (DBB)) were involved in the “response to stimulus” and “photomorphogenesis” processes in the intra-group comparisons of the GFD–T (Figure 3E,F). Additionally, nine genes (*LOC_Os02g52670* (ERF), *LOC_Os08g36920* (ERF), *LOC_Os01g58420* (ERF), *LOC_Os01g60640* (WRKY), *LOC_Os02g26430* (WRKY), *LOC_Os11g02520* (WRKY), *LOC_Os09g28310* (bZIP), *LOC_Os02g52780* (bZIP), and *LOC_Os01g53220* (HSF)) were involved in the “ET signaling pathway”, “response to stimulus”, “ABA signaling pathway”, “response to salt stress”, “response to water deprivation”, and “response to ABA” processes in the intra-group comparisons of the GFD–D (Figure 3G–I). The GO enrichment analysis showed that the DE-TF genes of T1 vs. T0 were mainly involved in the “ET signaling pathway”, ‘ABA signaling pathway”, and “photomorphogenesis” (Figure 3D). As the duration of the stress increased, additional biological processes, like the “response to stimulus” and “JA mediated signaling pathway”, were recruited (Figure 3E). And the process of “response to gibberellin (GA)” occurred at the recovery stage in RT vs. T0 (Figure 3F). In contrast, the DE-TF genes of D1 vs. D0 were involved in more additional biological processes, such as the “response to stimulus”, “stomatal complex development”, “response to water deprivation”, and “cell differentiation”, except the “ABA signaling pathway” (Figure 3G). In D2 vs. D0, the “ABA signaling pathway”, “response to GA”, and “response to salt stress” processes were activated (Figure 3H). However, more complex biological processes were found in the recovery period than saline stress in the intra-group comparison of the GFD–D (Figure 3I). All biological processes involved in each pairwise comparison are listed in Supplementary Table S5. Taken together, the GFD–T was involved in fewer biological processes during saline stress and recovery than the GFD–D, which may correspond to its phenotype being less severely damaged by NaCl. Remarkably, we found that the GFD–T differed from the GFD–D in that it activated the ABA signaling pathway in the early stage of saline stress.

### 2.4. Identification of Differentially Expressed TFs and Corresponding Gene Ontology Enrichment in GFD–T Compared to GFD–D

**Figure 4:**
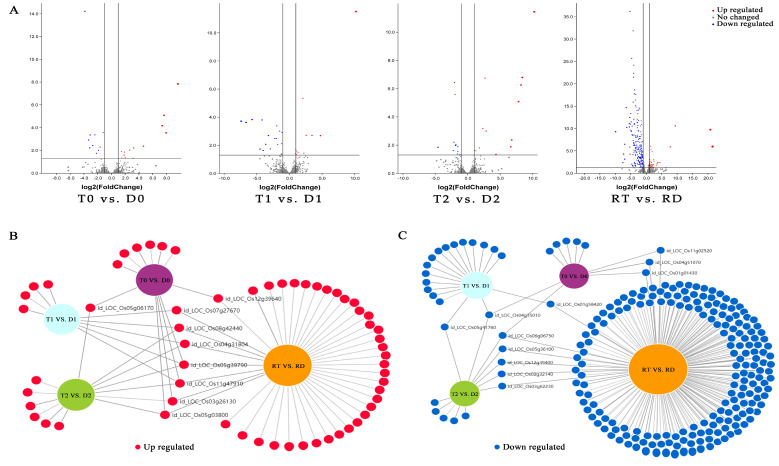

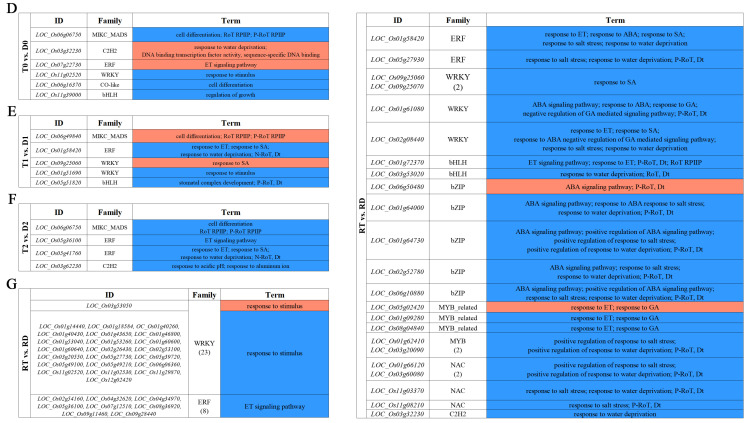
Identification of DE-TFs and gene functional characteristics between GFD–T and GFD–D. (**A**) Volcano plots of differentially expressed transcription factors (DE-TFs) in GFD–T relative to GFD–D at different time points. DiVenn analysis of (**B**) upregulated and (**C**) downregulated unique or shared DE-TFs among T0 vs. D0, T1 vs. D1, T2 vs. D2, and RT vs. RD. Red, blue, and gray dots indicate upregulated, downregulated, and non-changed genes, respectively. (**D**–**G**) Gene Ontology enrichment of DE-TFs derived from inter-group comparisons in GFD–T and GFD–D. The upregulated and downregulated genes involved in biological processes were marked in red and blue, respectively. D0/T0: diploid or tetraploid seedlings without NaCl stress; D1/T1: diploid or tetraploid seedlings treated with NaCl for 3 h; D2/T2: diploid or tetraploid seedlings treated with NaCl for 24 h; RD/RT: recovery culture of diploid or tetraploid seedlings for 24 h. DE-TFs: differentially expressed transcription factors; ABA: abscisic acid−activated; ET: ethylene−activated; SA: salicylic acid; GA: gibberellin; N−RoT, Dt: negative regulation of transcription, DNA−templated; P−RoT RPⅡP: positive regulation of transcription from RNA polymerase II promoter; P−RoT, Dt: positive regulation of transcription, DNA−templated; RoT RPⅡP: regulation of transcription from RNA polymerase II promoter; RoT, Dt: regulation of transcription, DNA−templated.

The overall DE-TFs between the GFD–T and GFD–D under the saline stress and recovery periods were compared (inter-group comparison) via volcano plots. There were 12 upregulated DE-TFs and 10 downregulated DE-TFs in the GFD–T compared to the GFD–D at 0 hpt (T0 vs. D0); 9 upregulated and 18 downregulated DE-TFs in the GFD–T compared to the GFD–D at 3 hpt (T1 vs. D1); 13 upregulated and 12 downregulated DE-TFs in the GFD–T compared to the GFD–D at 24 hpt (T2 vs. D2); as well as 37 upregulated and 194 downregulated DE-TFs in the GFD–T compared to the GFD–D at 24 hpr (RT vs. RD) (Figure 4A and Supplementary Table S6). To explore the up- or downregulated DE-TF genes that may play important roles in tetraploid salt tolerance, we used DiVenn analysis to elucidate the shared DE-TFs that were recurrent among the above comparison groups. A total of nine DE-TF genes were shared among the different comparison groups, of which four genes (*LOC_Os08g42440*, *LOC_Os004g31804*, *LOC_Os03g26130*, and *LOC_Os05g03800*) were shared among three comparison groups, and two genes (*LOC_Os055g39790* and *LOC_Os11g47910*) were shared among four comparison groups (Figure 4B). In addition, there were eleven downregulated DE-TFs shared among different comparisons, including two DE-TFs (*LOC_Os04g35010* and *LOC_Os06g06750*) shared among three comparison groups (Figure 4C). Among the identified co-expressed TFs, four genes (*LOC_Os06g06750* (MIKC_MADS), *LOC_Os11g02520* (WRKY), *LOC_Os01g58420* (ERF), and *LOC_Os05g36100* (ERF)) were involved in “cell differentiation”, “response to stimulus”, “response to ET”, “response to salicylic acid (SA)”, “response to water deprivation”, and “ET signaling pathway”, respectively (Figure 4D–G). The GO enrichment analysis showed that the most abundant biological processes occurred in RT vs. RD (Figure 4D–G). Interestingly, most of the DE-TFs involved in biological processes in each comparison group were downregulated genes (Figure 4D–G). Notably, the gene of *LOC_Os03g32230* (C2H2) was upregulated in T0 vs. D0, while it was downregulated in RT vs. RD (Figure 4D,G). And one gene (*LOC_Os09g25060*) belonging to the WRKY family was upregulated in T1 vs. D1, while it was downregulated in RT vs. RD (Figure 4E,G).

### 2.5. Functional Alterations of Core Differentially Expressed TFs in GFD–T and GFD–D

**Figure 5:**
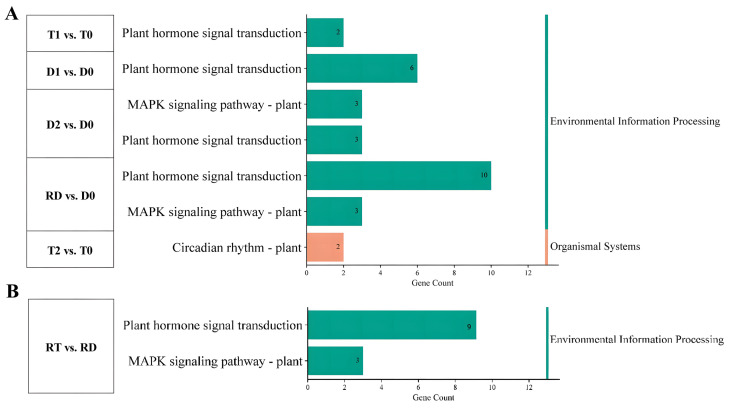
Functional analysis of DE-TFs in pairwise comparisons of GFD–T and GFD–D during saline stress and recovery stage. KEGG pathways of (**A**) intra-group comparison and (**B**) inter-group comparison in GFD–T and GFD–D. D0/T0: diploid or tetraploid seedlings without NaCl stress; D1/T1: diploid or tetraploid seedlings treated with NaCl for 3 h; D2/T2: diploid or tetraploid seedlings treated with NaCl for 24 h; RD/RT: recovery culture of diploid or tetraploid seedlings for 24 h. DE-TFs: differentially expressed transcription factors.

KEGG enrichment analysis was carried out to clarify the signaling pathways of the TF genes that participated in the above biological processes. The results showed that the DE-TFs from the intra-group comparison of GFD–T mainly participated in “plant hormone signal transduction” and “circadian rhythm” during saline stress, while the DE-TFs from the GFD–D were involved in “plant hormone signal transduction” and “MAPK signaling pathway” (Figure 5A and Supplementary Table S7). Two unique genes (*LOC_Os02g10860* and *LOC_Os08g06110*) derived from “circadian rhythm” were specifically downregulated in T2 vs. T0 (Figure 5A). Furthermore, the MAPK signaling pathway was identified in RT vs. RD (Figure 5B), which included three downregulated genes *(LOC_Os05g39720*, *LOC_Os05g27730*, and *LOC_Os01g61080*). However, these three identified genes derived from the MAPK signaling pathway were upregulated in the GFD–D intra-group comparisons (Figure 5A). These data suggested that GFD–T responds to saline stress in a different manner than GFD–D, as it turned on the circadian rhythm pathway.

**Figure 6:**
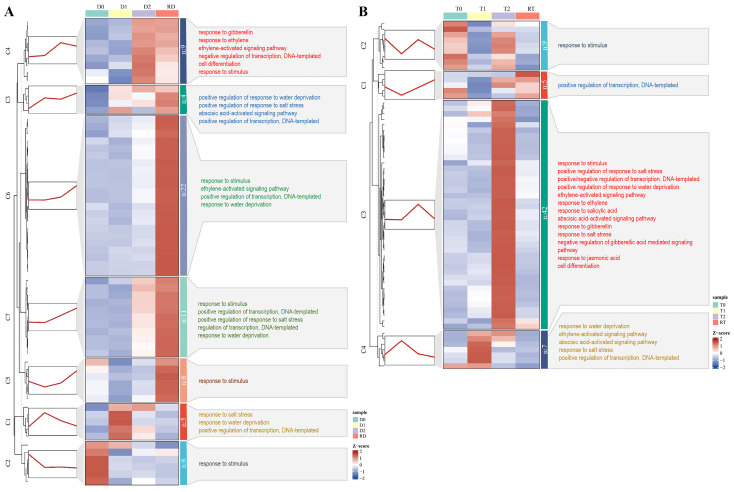
Clustering trend analysis of DE-TFs derived from the intra-group comparisons using k–mean algorithm. Gene cluster heatmaps and trend plots of (**A**) GFD–D and (**B**) GFD–T illustrate the different expression patterns. The representative genes of each cluster and the corresponding GO annotations are displayed on the left and right sides of the heatmap, respectively. DE-TFs: differentially expressed transcription factors. D0/T0: diploid or tetraploid seedlings without NaCl stress; D1/T1: diploid or tetraploid seedlings treated with NaCl for 3 h; D2/T2: diploid or tetraploid seedlings treated with NaCl for 24 h; RD/RT: recovery culture of diploid or tetraploid seedlings for 24 h.

To gain a more precise grasp of the dynamic expression trends of the DE-TF genes at different time points, we conducted a clustering trend analysis on the core DE-TF genes from the intra-group and inter-group comparisons in the GFD–T and GFD–D using the k–means algorithm. In the intra-group comparison of the GFD–D, 63 DE-TFs were grouped into seven clusters (C1–C7), and most of the 6 genes in C2, 5 genes in C1, 9 genes in C4, and 39 genes in C5–C7 were highly expressed at D0, D1, D2, and RD, respectively. Five (C3–C7) out of seven clusters exhibited an increased trend, especially as RD was significantly higher than D0 (Figure 6A). By contrast, 63 DE-TFs were grouped into four clusters (C1–C4) in the GFD–T, and most of the 9 genes in C2, 7 genes in C4, 42 genes in C3, and 5 genes in C1 were highly expressed at T0, T1, T2, and RT, respectively. Unlike the GFD–D, the expression levels of most of the DE-TF genes in the GFD–T at RT are comparable to those at T0 (Figure 6B). Observing the overall gene expression levels of the GFD–T and GFD–D, we found that most of the upregulated genes were obviously expressed at 24 hpt in the GFD–T, while they were significantly expressed at 24 hpr in the GFD–D, both of which were accompanied by more complex biological processes (Figure 6).

### 2.6. Dynamic Gene Network Analysis of GO Enrichment-Derived Core Genes

**Figure 7:**
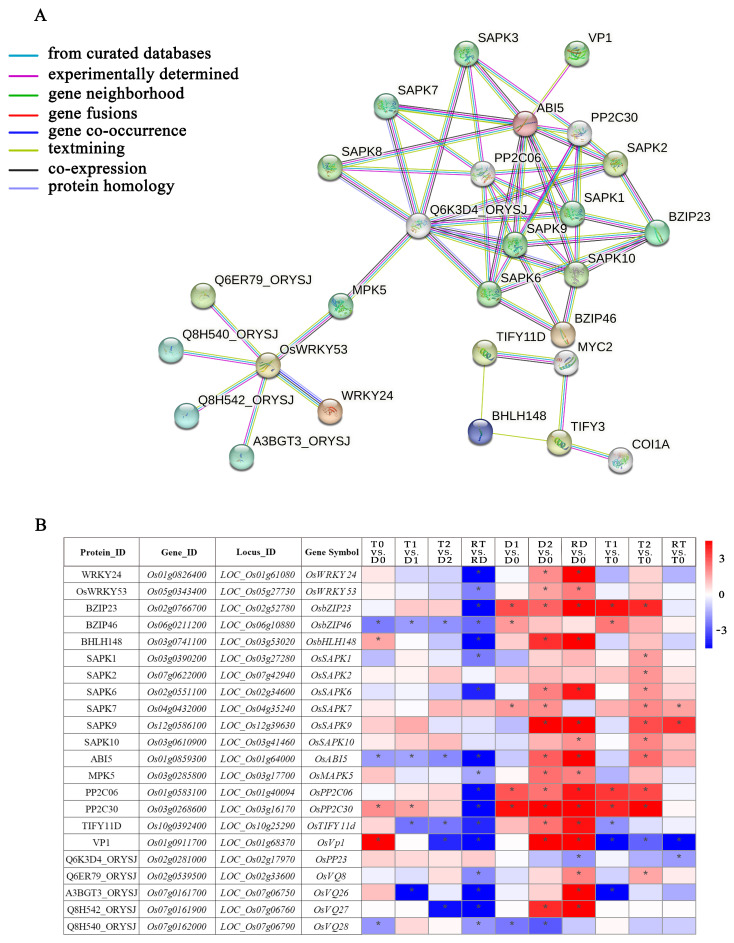
Family classification of core DE-TFs and dynamic gene network analysis. (**A**) Gene co-expressed network. Genes contained in significant Gene Ontology (GO) terms were analyzed and identified via a gene co-expression network using the STRING v11.0 database, with a high confidence score (≥0.9). (**B**) Heatmap analysis of core DE-TFs from dynamic gene network comparisons of GFD–T and GFD–D during saline stress and recovery period. Expression scores are shown as log2 fold change (FC) with * *p* < 0.05. Genes contained in significant Gene Ontology (GO) terms were analyzed and identified via gene co-expression network using the STRING v11.0 database, with a high confidence score (≥0.9). D0/T0: diploid or tetraploid seedlings without NaCl stress; D1/T1: diploid or tetraploid seedlings treated with NaCl for 3 h; D2/T2: diploid or tetraploid seedlings treated with NaCl for 24 h; RD/RT: recovery culture of diploid or tetraploid seedlings for 24 h. DE-TFs: differentially expressed transcription factors.

There were 108 core DE-TF genes in fourteen families derived from the GO enrichment analysis (Supplementary Table S8). Among them, WRKY and ERF were the two families with the highest numbers of DE-TFs (Supplementary Figure S3). From the perspective of the gene expressions across all the TF families, we found that more than 80% of the DE-TF genes showed an obvious downregulated trend in RT vs. RD, while they showed an obvious upregulated trend in RD vs. D0. Conversely, the DE-TFs in the GATA family showed upregulation and downregulation trends in RT vs. RD and RD vs. D0, respectively. In addition, all of the DE-TF genes in the HSF family were upregulated in the intra-group comparisons of the GFD–T and GFD–D (Supplementary Figure S3). To clarify the network regulation of the hub genes and further narrow down the dataset of genes in diverse biological processes for adaption to saline stress, core DE-TF genes derived from GO enrichment analysis were selected and analyzed via the STRING database to explore their mutual relationships. This network mainly consists of data interacting between 27 nodes and 51 edges (Figure 7A). We identified three families of TFs, including 5 genes (*OsWRKY24*, *OsWRKY53*, *OsbZIP23*, *OsbZIP46*, *OsbHLH148*) and 22 predicted binding genes. Among them, bHLH148 TFs were found to be actively interacting with TIFY TFs; WRKY53 interacted largely with MPK5, WRKY24, Q6ER79, Q8H540, Q8H542, and A3BGT3; bZIP mainly interacted with SAPK and indirectly interacted with other proteins, such as PP2C, VP1, Q6K3D4, etc. We further found six genes (*OsbZIP23*, *OsbZIP46*, *OsMPK5*, *OsbHLH148*, *OsTIFY11D*, and *OsCOI1A*) that play crucial roles in the regulation of salt or drought tolerance, some of which through the JA signaling or ABA-dependent regulation pathway (Figure 7A) [34,35,36,37]. The functions of the proteins represented in the interaction network are listed in Supplementary Table S9.

To elucidate the expression levels of the DE-TFs involved in the network in different comparisons, a heatmap with significantly expressed genes was generated (Figure 7B). The results showed that 17 of the 22 DE-TFs had significant downregulation in RT vs. RD. Among them, three DE-TFs (*OsbZIP46*, *OsABI5*, and *OsTIFY11D*) showed significant downregulation trends during the saline stress and recovery periods in the GFD–T versus the GFD–D. Different from the GFD–D, *OsSAPK1*, *OsSAPK2*, *OsSAPK10*, and *OsVQ8* were significantly upregulated in T2 vs. T0 of the GFD–T. Strikingly, the vacuolar H^+^–pyrophosphatase (H^+^–PPase, VP) *OsVP1* was obviously upregulated in T0 vs. D0. Moreover, it was downregulated during the saline stress and recovery periods in the GFD–T, whereas it was upregulated in the GFD–D at 24 h of the saline stress and recovery period. In addition, *OsbHLH148* was significantly upregulated in T0 vs. D0, and it was upregulated in the GFD–D at 24 h of the recovery period, but not in the GFD–T. *OsPP2C30* was also significantly upregulated in T0 vs. D0, but there was no significant change in the RT vs. T0 of the GFD–T, unlike in the GFD–D. Taken together, the expression levels of the TFs in the diploid and tetraploid rice at different stages of stress were significantly different, and it was especially during the recovery period in the diploid rice that the expression levels of most of the TFs significantly changed, which implies that diploid rice needs to activate considerably more TFs than tetraploid rice to rescue it from salt poisoning.

## 3. Discussion

In agricultural production, it is well known that abiotic stresses, such as saline stress, are major disturbances that negatively affect crop performances. Plant defense mechanisms against stress have been studied at the molecular level for many years to identify the main factors that enhance plant tolerance [38]. Noticeably, enhancing stress tolerance in plants by manipulating the expressions of TF-encoding genes has become a popular approach, as TFs act as nuclear effector factors in multiple signal cascades to provide nodes for information integration, forming an elaborate regulatory layer, typically involving post-translational modifications and the modulation and coordination of activities [39]. Until now, studies on TFs that regulate abiotic stress responses have mostly focused on single TFs and their isolated functions [40]. However, the understanding of TF regulators and the gene network control mechanisms of their interactions is still inadequate.

Salinity affects leaf growth in both the short and long terms, and the causes of stunted growth differ [41,42]. Saline stress is first sensed by the roots and impairs plant growth in the short term through osmotic stress caused by decreased water availability, and it impairs plant growth in the long term through salt-induced ionic toxicity caused by nutrient imbalances in the cytosol [43]. Although shoot ion toxicity only becomes apparent in later stages, Na^+^-specific responses have been reported to occur in roots during the early stages (<24 h) of saline stress exposure [44]. Therefore, in the present study, we investigated the responses of diploid and tetraploid rice plants in the early stages (3 hpt and 24 hpt) of saline stress and recovery period (24 hpr). We found that the autotetraploid rice (GFD–T) exhibited a superior salt-tolerance phenotype compared to its diploid ancestor (GFD–D) (Figure 1). In order to explore the regulatory network mechanisms behind the salt tolerance, we performed a transcriptome-based comparative analysis of the TFs between the salt-tolerant GFD–T and salt-sensitive GFD–D in NaCl stress and recovery periods. The clustering analysis of the global TF gene expression showed that the overall expression levels of genes from 56 TF families in the GFD–T during the recovery period were similar to those of the control group (Figure 2). More than that, the number of DE-TFs expressed during the recovery period of the GFD–T (RT vs. T0) was decreased compared with that expressed during saline stress at 24 hpt (T2 vs. T0), while the number of DE-TFs in RD vs. D0 increased significantly during the recovery period of the GFD–D (Figure 3A). In general, the mildly stressed plants recovered faster after the stress was alleviated, while the plants that were subjected to severe water stress recovered only 40–60% of the maximum photosynthetic rate within a day after rewatering. Previous studies have shown that the relative electrolyte leakage of peanut leaves under recovery conditions after saline stress did not decrease, or even increased, indicating that the additional stress damaged the stability of the cell membrane [45]. Therefore, our results may suggest that the saline stress caused relatively less damage to the GFD–T, which maintained the stability of its cell membrane and enabled it to quickly return to the initial state after a recovery period. Furthermore, one DE-TF gene (*LOC_Os02g32590*, *OsHsfA3*) that belongs to the HSF family was co-expressed among T1 vs. T0/D1 vs. D0_common, T2 vs. T0/D2 vs. D0_common, and RT vs. T0/RD vs. D0_common (Figure 3B), which indicates that *OsHsfA3* may play an important role in both GFD–T and GFD–D during the saline stress and recovery periods. For instance, *OsHsfA3* plays a positive regulatory role in rice drought resistance by increasing the ABA levels and regulating the polyamine levels to maintain ROS homeostasis [46]. As we know, salinity and drought share a physiological water deficit, and plants respond to hyper-ionic and hyper-osmotic stresses in addition to dehydration under prolonged saline stress [47]. Moreover, bZIP23 and bZIP46 conferred drought or salinity tolerance through an ABA-dependent regulation pathway [34,35]. In this study, the GFD–T activated the ABA signaling pathway by interacting with bZIPs (*bZIP23* or *bZIP72*) during the early stages of saline stress (Figure 3D). *bZIP72* is a key regulator of abiotic stress responses and ABA signaling transduction pathways, which play a key role in the ABA-mediated salt- and drought-tolerance pathways in rice [48].

However, saline stress affects more extensive genes, and more intensively, compared with drought stress, which may reflect the combined effects of dehydration and osmotic stress in saline-treated plants [45]. In other words, more intense stress may trigger a more complex regulatory network. In this study, the GO enrichment analysis showed that more biological processes were activated in the pairwise comparisons of the GFD–D than in the GFD–T (Figure 3D–I), and most of the DE-TF genes were negatively regulated in the GFD–T relative to the GFD–D (Figure 4D–G). The clustering-trend analysis results represented that most of the upregulated genes involved in complex biological processes were clustered at 24 hpt and 24 hpr in the GFD–T and GFD–D, respectively (Figure 6). These results, combined with the phenotypic data overall, reflected that the GFD–D was damaged by saline stress to a greater extent than the GFD–T, thereby triggering more complex biological strategies to cope with it. Moreover, a unique circadian rhythm pathway was identified in T2 vs. T0 of the GFD–T via KEGG enrichment analysis (Figure 5A). This pathway included a downregulated gene (*LOC_Os02g10860* (*OsbZIP18*)) that negatively controls the UV–B tolerance in rice [49]. A previous study indicated that *UVR8*-overexpressing plants showed more severe growth defects under osmotic and salt stress, while the inactivation of *UVR8* expression enhanced the shoot growth under high-salt stress [50]. These clues implied that significantly reducing the expression of *OsbZIP18* in the GFD–T may have endowed it with stronger salt tolerance in our study (Supplementary Figure S3). Additionally, the MAPK signaling pathway was identified in RT vs. RD, which included three downregulated genes (*OsWRKY24*, *OsWKKY53*, *OsWKKY70*). In contrast, the above three identical genes derived from the MAPK signaling pathway were upregulated in the GFD–D intra-group comparisons (Figure 5B). In *Arabidopsis*, *AtWRKY70* acted as a negative regulator in abiotic stress signaling, with a similar result obtained in tomato [51,52]. The inhibition of *PsnWRKY70* expression could enhance the saline tolerance in *P. simonii × P. nigra* [53]. Therefore, the *OsWRKYs* identified in this study could provide new clues for exploring the mechanism of saline tolerance in rice.

In common cells or organisms, physiological and biological processes, including cellular interactions, as well as developmental control and metabolic processes, are regulated by PPIs between different TFs [54]. To explore the network of hub genes related to salt tolerance, we analyzed the network of 108 core TF genes and highlighted many hub genes derived from the WRKY, bZIP, bHLH, SAPK, ABI, VP, etc., families (Figure 7 and Supplementary Figure S3). These hub genes may perform key functions in saline defense. An interesting phenomenon is that a single gene can be conferred with multiple forms of stress (drought, salt, cold, heat, etc.) tolerance, such as the bHLH148/TIFY11D/COI1A complex, which may act on an initial response of jasmonate-regulated gene expression to drought tolerance, and the knockout mutants of the *Arabidopsis* ortholog gene *bHLH148*, which showed a loss of function for drought and salt-tolerance phenotypes [36]. The “*SAPK10-WRKY87-ABF1*” module synergistically interacts to improve drought and salt tolerance in rice [55]. The overexpression of *OsHSP18.6* results in increased thermotolerance and displays general tolerance to heat, drought, salt, and cold [56]. And the co-overexpressed *AVP1* and *OsSIZ1* in *Arabidopsis* substantially enhance the plant tolerance to drought, salt, and heat stresses [57]. However, the mechanism of this multiple tolerance has not been fully elucidated. Based on previous studies, most genes with multiple forms of stress are involved in the synthesis of osmolytes or are chaperones, suggesting that different stresses induce similar damage in response to osmotic and/or oxidative stress [58] and maintain the stability of the cellular environment by activating similar signaling pathways under stress [59]. In this study, *OsSAPK1*, *OsSAPK2*, *OsSAPK10*, and *OsVQ8* were significantly upregulated in the GFD–T but not in the GFD–D at 24 hpt. *SAPK1* and *SAPK2* may function collaboratively as positive regulators of saline stress tolerance to synergistically reduce Na^+^ toxicity by influencing the distribution of Na^+^ between the shoots and roots, Na^+^ exclusion from the cytoplasm, and Na^+^ sequestration into the vacuole [60]. In addition, the “miR2105-(OsSAPK10)-OsbZIP86-OsNCED3” module has a potential role to play in improving drought tolerance in rice [61]. The WRKY10-VQ8 module regulated rice thermotolerance, and *SsVQ8* was negatively induced via cold stress in sugarcane [62,63]. Based on the above information, we speculate that *OsSAPK1*, *OsSAPK2*, *OsSAPK10*, and *OsVQ8* may have a positive effect on the salt tolerance of GFD–T. However, further studies should be performed. Furthermore, *OsbHLH148* was significantly upregulated in the GFD–T versus the GFD–D at 0 hpt, and it was significantly upregulated in the GFD–D at 24 h of the recovery period, but not in the GFD–T; however, there were no significant differences in the GFD–T versus the GFD–D at 3 hpt or 24 hpt. Similarly, *OsVP1* was upregulated in the GFD–T versus the GFD–D at 0 hpt (Figure 7B). Because VPs mediate vacuolar Na^+^ absorption, which is an important process for plant salt tolerance [64], these data suggest that the significant increase in the expression levels of *OsbHLH148* and *OsVP1* after chromosome doubling in tetraploid rice may give it greater advantages in alleviating physiological water deficit and reducing salt toxicity, respectively [47,64]. In addition, MPK5 acted as a positive regulator of drought and salt tolerance [37]. Previous studies have shown that WRKY proteins constitute a critical link between the MAPK signaling pathways and downstream transcriptional reprogramming. This study exhibited a highly interconnected hub of WRKY24 and WRKY53 that interacted with MPK5, which suggested that WRKY53 may play an integral role in the salt-tolerant regulatory network of MPK5 (Figure 7A). Furthermore, the repression of *ABI5* (*bZIP10*) promoted salt tolerance [65]. In this study, *OsABI5* was significantly downregulated in the GFD–T versus the GFD–D at 0 hpt, and it showed a significant downregulation trend during the saline stress and recovery periods in the GFD–T versus the GFD–D, suggesting that the low expression level of *OsABI5* in tetraploid rice may play an important role in its salt tolerance. *OsSAPK10* and *OsVQ8* were significantly upregulated in T2 vs. T0 of the GFD–T.

## 4. Materials and Methods

### 4.1. Plant Materials and NaCl Treatments

Diploid (GFD–D) and tetraploid rice (GFD–T) plants of *Oryza sativa* L. ssp. *japonica* were used in the present study. The GFD–T was a natural mutant autotetraploid rice derived from GFD–D screened in the field, and self-pollinated for six generations. Two weeks after seed germination, seedlings at the trefoil stage were treated with a series of 0 mM, 50 mM, 100 mM, 125 mM, 150 mM, and 200 mM saline solutions (NaCl dissolved in Kimura B solution [66]) for 24 h, followed by the removal of saline stress and recovery culture in the Kimura B solution for 24 h to determine optimal concentrations. An optimal concentration of 125 mM NaCl was screened via phenotyping and used in subsequent saline stress experiments. The composition of the Kimura B solution was as follows: 0.09 mM KH_2_PO_4_; 0.27 mM MgSO_4_; 0.18 mM (NH_4_)_2_SO_4_; 0.09 mM KNO_3_; 0.18 mM Ca(NO_3_)_2_; 3 μM H_3_BO_3_; 0.5 μM MnCl_2_; 1 μM (NH_4_)_6_Mo_7_O_24_; 0.4 μM ZnSO_4_; 0.2 μM CuSO_4_; and 20 μM Fe (III)-EDTA, and it was adjusted to pH 4.5–5.6 for the preparation of the saline solution and recovery culture. The experimental progress was conducted in a growth chamber with 16 h light (200 µmol m^−2^ s^−1^, 28 °C) and 8 h darkness (25 °C) with 70% relative humidity at the College of Agronomy of Jilin Agricultural University (Changchun, China). Ninety GFD–D and ninety GFD–T seedlings with representative and vigorous conditions from three replicates were selected and exposed to the stress for phenotyping. Among them, three individual plants each from the GFD–D and GFD–T for RNA-seq analysis were selected for collecting samples at 0 h, 3 h, and 24 h after stress and 24 h after recovery, labeled as T0, T1, T2, and RT in the GFD–T, and D0, D1, D2, and RT in the GFD–D. Therefore, a total of twenty-four RNA samples for RNA sequencing (RNA-seq) were collected, which were immediately frozen in liquid nitrogen and stored at −80 °C for further analysis.

### 4.2. RNA Isolation, RNA-seq, and qRT–PCR Validation

Total RNA of rice seedlings was isolated using the TRIzol method (Life Technologies Invitrogen, Carlsbad, CA, USA) according to the manufacturer’s protocol. The RNA purity, concentration, and integrity were assessed with NanoDrop 2000 (Thermo Scientific, Waltham, MA, USA), a Qubit 2.0 Fluorometer (Life Technologies, Carlsbad, CA, USA), and the Agilent 2100 Bioanalyzer system, respectively. Qualified RNA was processed for RNA-seq to construct the RNA-seq library, which was performed on the Illumina sequencing platform by Biomarker Technologies Co., Ltd. (Beijing, China). Raw image data generated via sequencing were transformed from base-calling to sequence data, which are called raw data/raw reads, and were stored in fastq format. Clean reads were obtained by removing low-quality sequences from raw data. High-quality reads were mapped to the *Oryza sativa* Nipponbare reference genome (MSUv7.0, http://rice.plantbiology.msu.edu/index.shtml, accessed on 5 November 2022) [67], using Hierarchical Indexing for Spliced Alignment of Transcripts (HISAT2, version 2.2.1) [68], which is open–source software freely available at https://github.com/DaehwanKimLab/hisat2 (accessed on 29 July 2023).

The qRT-PCR-based expression analysis was used to perform validation for seven genes. The qPCR Primer Database (https://qprimerdb.biodb.org/, accessed on 4 January 2023) was used for the primer design (Supplementary Table S1). cDNAs were synthesized from approximately 2 μg of total RNA using First-Strand cDNA Synthesis SuperMix (TransGen Biotech, Beijing, China) for qPCR. qRT-PCR amplification was performed in a volume of 20 μL containing 10 μL of 2× TransStart^®^ Top Green qPCR SuperMix using a Top Green qPCR SuperMix kit (TransGen Biotech, Beijing, China). Each PCR cycle was conducted as follows: 94 °C for 10 s, 55 °C for 10 s, and 72 °C for 10 s, followed by 40 cycles. All reactions were performed in triplicate, and the relative gene expression levels were calculated using the 2^−ΔΔCt^ method [69], which is a convenient method to analyze relative changes in gene expression from qRT-PCR experiments.

### 4.3. Transcriptome Assembly and Functional Annotation

De novo assembly was performed in StringTie [70]. Unigenes were identified based on the Plant Transcription Factor Database (PlantTFDB v5.0, http://planttfdb.gao-lab.org/, accessed on 2 June 2023). The functions of the differentially expressed TFs were examined via Gene Ontology (GO) and Kyoto Encyclopedia of Genes and Genomes (KEGG) analyses using the Database for Annotation, Visualization, and Integrated Discovery (DAVID) [71]. All false discovery rate (FDR)-adjusted *p*-values (*q*-values) with *q* < 0.05 were treated as significant.

### 4.4. Co-Expression Analysis of the Differentially Expressed TFs

Gene expression levels or transcripts were measured according to FPKM (fragments per kilobase of transcript per million fragments mapped) in StringTie using the maximum-flow algorithm. The genes with count values > 30 were detected for differentially expressed genes (DEGs) with *q*-values < 0.05 and |log2 (fold changes)| ≥ 1, and plotted using TBtools (version 1.09866). The Venn diagrams were generated via a web-based tool (Venny 2.1 at https://bioinfogp.cnb.csic.es/tools/venny/ (accessed on 10 July 2023)), and the DiVenn analysis was performed online at https://divenn.tch.harvard.edu/ (accessed on 15 July 2023). The volcano plots were generated using TBtools. The upset plots were built with the R package UpSetR (version 1.4.0, https://cran.r–project.org/web/packages/UpSetR/index.html, accessed on 22 May 2023). A protein–protein interaction (PPI) analysis was performed with the STRING database (version 12.0, http://string-db.org, accessed on 22 July 2023) as the background interactions. The parameter-of-confidence score was set to 0.9 for the filter of the network. In the STRING network, nodes represented the proteins generated by a single protein-coding gene locus, and edges represented PPIs containing known and predicted interactions. The k-means algorithm clustering analysis was conducted using the factoextra R package and online platform for data analysis and visualization (https://www.bioinformatics.com.cn, accessed on 25 July 2023).

### 4.5. Statistical Analysis

The heatmaps for the gene expression levels of the global TFs and log2 (fold-change) values of differentially expressed TFs derived from different comparisons were visualized using the TBtools and pheatmap package in R [72] (version 1.0.12, https://cran.r–project.org/web/packages/pheatmap/index.html, accessed on 5 May 2023), respectively. Other data visualization was carried out in Microsoft Office Excel Version 2307 (Build 16827.20166).

## 5. Conclusions

This study performed a transcriptome-based comparative analysis of the TF families in salt-tolerant GFD–T and salt-sensitive GFD–D during NaCl stress and recovery periods. Our results showed that the GFD–T had superior phenotypic characteristics and an intrinsic transcriptional regulation ability compared with the GFD–D during the early stage of saline stress and recovery period, mainly manifested in the fact that its overall gene expression levels were comparable to those of the control during the recovery period, which may have been due to its rapid activation of the ABA signaling pathway and the initiation of a specific circadian rhythm process, resulting in a unique gene regulatory network for GFD–T to resist saline stress. However, further work is necessary to verify these new findings. In addition, some core DE-TFs (such as *OsbZIP23*, *OsbHLH148*, *OsABI5*, *OsVP1*, *OsSAPK1*, *OsSAPK2*, *OsSAPK10*, and *OsVQ8*, etc.) were found that may have potential roles to play in tetraploid salt tolerance. The systematic identification and classification of the TF genes in this study are essential for understanding the evolutionary directions, biological roles, and regulatory networks of polyploid rice.

## Data Availability

The datasets generated and analyzed in this study are available at PRJNA1010456 (https://www.ncbi.nlm.nih.gov/bioproject/PRJNA1010456, accessed on 30 August 2023).

## Supplementary Materials

The supporting information can be downloaded at https://www.mdpi.com/article/10.3390/ijms242115982/s1.

## References

[1] Zhang R., Wang Y., Hussain S., Yang S., Li R., Liu S., Chen Y., Wei H., Dai Q., Hou H. (2022). Study on the effect of salt stress on yield and grain quality among different rice varieties. Front. Plant Sci..

[2] Salgotra R.K., Chauhan B.S. (2023). Ecophysiological responses of rice (*Oryza sativa* L.) to drought and high temperature. Agronomy.

[3] Rao P.S., Mishra B., Gupta S.R. (2013). Effects of soil salinity and alkalinity on grain quality of tolerant, semi-tolerant and sensitive rice genotypes. Rice Sci..

[4] Fawcett J.A., Maere S., Van de Peer Y. (2009). Plants with double genomes might have had a better chance to survive the cretaceous–tertiary extinction event. Proc. Natl. Acad. Sci. USA.

[5] Van de Peer Y., Ashman T.L., Soltis P.S., Soltis D.E. (2021). Polyploidy: An evolutionary and ecological force in stressful times. Plant Cell.

[6] Wang L., Cao S., Wang P., Lu K., Song Q., Zhao F.J., Chen Z.J. (2021). DNA hypomethylation in tetraploid rice potentiates stress-responsive gene expression for salt tolerance. Proc. Natl. Acad. Sci. USA.

[7] Wang N., Wang S., Qi F., Wang Y., Lin Y., Zhou Y., Meng W., Zhang C., Wang Y., Ma J. (2022). Autotetraploidization gives rise to differential gene expression in response to saline stress in rice. Plants.

[8] Nakano T., Suzuki K., Fujimura T., Shinshi H. (2006). Genome-wide analysis of the ERF gene family in *Arabidopsis* and rice. Plant Physiol..

[9] Chen H., Yu H., Jiang W., Li H., Wu T., Chu J., Xin P., Li Z., Wang R., Zhou T. (2021). Overexpression of ovate family protein 22 confers multiple morphological changes and represses gibberellin and brassinosteroid signalings in transgenic rice. Plant Sci..

[10] Hu H., You J., Fang Y., Zhu X., Qi Z., Xiong L. (2008). Characterization of transcription factor gene SNAC2 conferring cold and salt tolerance in rice. Plant Mol. Biol..

[11] Ma Q., Dai X., Xu Y., Guo J., Liu Y., Chen N., Xiao J., Zhang D., Xu Z., Zhang X. (2009). Enhanced tolerance to chilling stress in OsMYB3R-2 transgenic rice is mediated by alteration in cell cycle and ectopic expression of stress genes. Plant Physiol..

[12] Hossain M.A., Cho J.I., Han M., Ahn C.H., Jeon J.S., An G., Park P.B. (2010). The ABRE-binding bZIP transcription factor OsABF2 is a positive regulator of abiotic stress and ABA signaling in rice. J. Plant Physiol..

[13] Park M.R., Yun K.Y., Mohanty B., Herath V., Xu F., Wijaya E., Bajic V.B., Yun S.J., De Los Reyes B.G. (2010). Supra-optimal expression of the cold-regulated OsMyb4 transcription factor in transgenic rice changes the complexity of transcriptional network with major effects on stress tolerance and panicle development. Plant Cell Environ..

[14] Mallikarjuna G., Mallikarjuna K., Reddy M.K., Kaul T. (2011). Expression of OsDREB2A transcription factor confers enhanced dehydration and salt stress tolerance in rice (*Oryza sativa* L.). Biotechnol. Lett..

[15] Teng Y., Lv M., Zhang X., Cai M., Chen T. (2022). BEAR1, a bHLH transcription factor, controls salt response genes to regulate rice salt response. J. Plant Biol..

[16] Huang J., Liu F., Chao D., Xin B., Liu K., Cao S., Chen X., Peng L., Zhang B., Fu S. (2022). The WRKY transcription factor OsWRKY54 is involved in salt tolerance in rice. Int. J. Mol. Sci..

[17] Yu Z., Duan X., Luo L., Dai S., Ding Z., Xia G. (2020). How plant hormones mediate salt stress responses. Trends Plant Sci..

[18] Leyser O. (2017). Auxin signaling. Plant Physiol..

[19] Yan F., Zhu Y., Zhao Y., Wang Y., Li J., Wang Q., Liu Y. (2020). De novo transcriptome sequencing and analysis of salt-, alkali-, and drought-responsive genes in *Sophora alopecuroides*. BMC Genom..

[20] Yin L., Zander M., Huang S.C., Xie M., Song L., Guzm N.J., Hann E., Shanbhag B.K., Ng S., Jain S. (2023). Transcription factor dynamics in cross-regulation of plant hormone signaling pathways. bioRxiv.

[21] Nemhauser J.L., Hong F., Chory J. (2006). Different plant hormones regulate similar processes through largely nonoverlapping transcriptional responses. Cell.

[22] Chang K.N., Zhong S., Weirauch M.T., Hon G., Pelizzola M., Li H., Huang S.S., Schmitz R.J., Urich M.A., Kuo D. (2013). Temporal transcriptional response to ethylene gas drives growth hormone cross-regulation in *Arabidopsis*. Elife.

[23] Clark N.M., Nolan T.M., Wang P., Song G., Montes C., Valentine C.T., Guo H., Sozzani R., Yin Y., Walley J.W. (2021). Integrated omics networks reveal the temporal signaling events of brassinosteroid response in *Arabidopsis*. Nat. Commun..

[24] Song L., Huang S.C., Wise A., Castanon R., Nery J.R., Chen H., Watanabe M., Thomas J., Bar-Joseph Z., Ecker J.R. (2016). A transcription factor hierarchy defines an environmental stress response network. Science.

[25] Zander M., Lewsey M.G., Clark N.M., Yin L., Bartlett A., Saldierna Guzmán J.P., Hann E., Langford A.E., Jow B., Wise A. (2020). Integrated multi-omics framework of the plant response to jasmonic acid. Nat. Plants.

[26] Natukunda M.I., Mantilla-Perez M.B., Graham M.A., Liu P., Salas-Fernandez M.G. (2022). Dissection of canopy layer-specific genetic control of leaf angle in sorghum bicolor by RNA sequencing. BMC Genom..

[27] Chen L., Dou P., Li L., Chen Y., Yang H. (2022). Transcriptome-wide analysis reveals core transcriptional regulators associated with culm development and variation in Dendrocalamus sinicus, the strongest woody bamboo in the world. Heliyon.

[28] Zhang X., Long Y., Chen X., Zhang B., Xin Y., Li L., Cao S., Liu F., Wang Z., Huang H. (2021). A NAC transcription factor OsNAC3 positively regulates ABA response and salt tolerance in rice. BMC Plant Biol..

[29] Huang S., Ma Z., Hu L., Huang K., Zhang M., Zhang S., Jiang W., Wu T., Du X. (2021). Involvement of rice transcription factor OsERF19 in response to ABA and salt stress responses. Plant Physiol. Biochem..

[30] Zhu N., Cheng S., Liu X., Du H., Dai M., Zhou D.X., Yang W., Zhao Y. (2015). The R2R3-type MYB gene *OsMYB91* has a function in coordinating plant growth and salt stress tolerance in rice. Plant Sci..

[31] Heenan D., Lewin L., McCaffery D. (1988). Salinity tolerance in rice varieties at different growth stages. Aust. J. Exp. Agric..

[32] Zeng L., Shannon M.C., Lesch S.M. (2001). Timing of salinity stress affects rice growth and yield components. Agric. Water Manag..

[33] Singh R.K., Kota S., Flowers T.J. (2021). Salt tolerance in rice: Seedling and reproductive stage QTL mapping come of age. Theor. Appl. Genet..

[34] Xiang Y., Tang N., Du H., Ye H., Xiong L. (2008). Characterization of OsbZIP23 as a key player of the basic leucine zipper transcription factor family for conferring abscisic acid sensitivity and salinity and drought tolerance in rice. Plant Physiol..

[35] Tang N., Zhang H., Li X., Xiao J., Xiong L. (2012). Constitutive activation of transcription factor OsbZIP46 improves drought tolerance in rice. Plant Physiol..

[36] Seo J.S., Joo J., Kim M.J., Kim Y.K., Nahm B.H., Song S.I., Cheong J.J., Lee J.S., Kim J.K., Choi Y.D. (2011). OsbHLH148, a basic helix-loop-helix protein, interacts with OsJAZ proteins in a jasmonate signaling pathway leading to drought tolerance in rice. Plant J..

[37] Sharma R., De Vleesschauwer D., Sharma M.K., Ronald P.C. (2013). Recent advances in dissecting stress-regulatory crosstalk in rice. Mol. Plant.

[38] Hoang X.L.T., Nhi D.N.H., Thu N.B.A., Thao N.P., Tran L.P. (2017). Transcription factors and their roles in signal transduction in plants under abiotic stresses. Curr. Genom..

[39] Pandey D.M., Mishra A.N. (2016). Transcription Factor: Current and Future Perspectives. Austin J. Biotechnol. Bioeng..

[40] Lindemose S., O’Shea C., Jensen M.K., Skriver K. (2013). Structure, function and networks of transcription factors involved in abiotic stress responses. Int. J. Mol. Sci..

[41] Munns R., Schachtman D., Condon A. (1995). The significance of a two-phase growth response to salinity in wheat and barley. Funct. Plant Biol..

[42] Fricke W., Akhiyarova G., Wei W., Alexandersson E., Miller A., Kjellbom P.O., Richardson A., Wojciechowski T., Schreiber L., Veselov D. (2006). The short-term growth response to salt of the developing barley leaf. J. Exp. Bot.

[43] Munns R. (2005). Genes and salt tolerance: Bringing them together. New Phytol..

[44] Deolu-Ajayi A.O., Meyer A.J., Haring M.A., Julkowska M.M., Testerink C. (2019). Genetic loci associated with early salt stress responses of roots. iScience.

[45] Cui F., Sui N., Duan G., Liu Y., Han Y., Liu S., Wan S., Li G. (2018). Identification of metabolites and transcripts involved in salt stress and recovery in peanut. Front. Plant Sci..

[46] Zhu M.D., Zhang M., Gao D.J., Zhou K., Tang S.J., Zhou B., Lv Y.M. (2020). Rice OsHSFA3 gene improves drought tolerance by modulating polyamine biosynthesis depending on abscisic acid and ROS levels. Int. J. Mol. Sci..

[47] Chaves M.M., Flexas J., Pinheiro C. (2009). Photosynthesis under drought and salt stress: Regulation mechanisms from whole plant to cell. Ann. Bot.

[48] Baoxiang W., Yan L., Yifeng W., Jingfang L., Zhiguang S., Ming C., Yungao X., Bo X., Bo Y., Jian L. (2021). OsbZIP72 is involved in transcriptional gene-regulation pathway of abscisic acid signal transduction by activating rice high-affinity potassium transporter OsHKT1;1. Rice Sci..

[49] Sun Y., Wang B., Ren J., Zhou Y., Han Y., Niu S., Zhang Y., Shi Y., Zhou J., Yang C. (2022). OsbZIP18, a positive regulator of serotonin biosynthesis, negatively controls the UV-B tolerance in rice. Int. J. Mol. Sci..

[50] Fasano R., Gonzalez N., Tosco A., Dal Piaz F., Docimo T., Serrano R., Grillo S., Leone A., Inze D. (2014). Role of *Arabidopsis* UV RESISTANCE LOCUS 8 in plant growth reduction under osmotic stress and low levels of UV-B. Mol. Plant.

[51] Abbasi S., Sadeghi A., Safaie N. (2020). *Streptomyces* alleviate drought stress in tomato plants and modulate the expression of transcription factors ERF1 and WRKY70 genes. Sci. Hortic.-Amst..

[52] Li J., Besseau S., Törönen P., Sipari N., Kollist H., Holm L., Palva E.T. (2013). Defense-related transcription factors WRKY70 and WRKY54 modulate osmotic stress tolerance by regulating stomatal aperture in *Arabidopsis*. New Phytol..

[53] Zhao H., Jiang J., Li K., Liu G. (2017). *Populus simonii* × *Populus nigra* WRKY70 is involved in salt stress and leaf blight disease responses. Tree Physiol..

[54] Jiang Z., Dong X., Zhang Z. (2016). Network-based comparative analysis of *Arabidopsis* immune responses to *Golovinomyces orontii* and *Botrytis cinerea* infections. Sci. Rep..

[55] Yan L., Baoxiang W., Jingfang L., Zhiguang S., Ming C., Yungao X., Bo X., Bo Y., Jian L., Jinbo L. (2021). A novel SAPK10-WRKY87-ABF1 biological pathway synergistically enhance abiotic stress tolerance in transgenic rice (*Oryza sativa*). Plant Physiol. Biochem..

[56] Wang A., Yu X., Mao Y., Liu Y., Liu G., Liu Y., Niu X. (2015). Overexpression of a small heat-shock-protein gene enhances tolerance to abiotic stresses in rice. Plant Breed..

[57] Esmaeili N., Yang X., Cai Y., Sun L., Zhu X., Shen G., Payton P., Fang W., Zhang H. (2019). Co-overexpression of AVP1 and OsSIZ1 in *Arabidopsis* substantially enhances plant tolerance to drought, salt, and heat stresses. Sci. Rep..

[58] Wang W., Vinocur B., Altman A. (2003). Plant responses to drought, salinity and extreme temperatures: Towards genetic engineering for stress tolerance. Planta.

[59] Zhu J.K. (2002). Salt and drought stress signal transduction in plants. Ann. Rev. Plant Biol..

[60] Lou D., Wang H., Yu D. (2018). The sucrose non-fermenting-1-related protein kinases SAPK1 and SAPK2 function collaboratively as positive regulators of salt stress tolerance in rice. BMC Plant Biol..

[61] Gao W., Li M., Yang S., Gao C., Su Y., Zeng X., Jiao Z., Xu W., Zhang M., Xia K. (2022). miR2105 and the kinase OsSAPK10 co-regulate OsbZIP86 to mediate drought-induced ABA biosynthesis in rice. Plant Physiol..

[62] Chen S., Cao H., Huang B., Zheng X., Liang K., Wang G.L., Sun X. (2022). The WRKY10-VQ8 module safely and effectively regulates rice thermotolerance. Plant Cell Environ..

[63] Liu Y., Liu X., Yang D., Yin Z., Jiang Y., Ling H., Huang N., Zhang D., Wu J., Liu L. (2022). A comprehensive identification and expression analysis of VQ motif-containing proteins in Sugarcane (*Saccharum spontaneum* L.) under phytohormone treatment and cold stress. Int. J. Mol. Sci..

[64] Meng L., Li S., Guo J., Guo Q., Mao P., Tian X. (2017). Molecular cloning and functional characterisation of an H+-pyrophosphatase from Iris lactea. Sci. Rep..

[65] Zou M., Guan Y., Ren H., Zhang F., Chen F. (2008). A bZIP transcription factor, OsABI5, is involved in rice fertility and stress tolerance. Plant Mol. Biol..

[66] Chen J., Huang X.Y., Salt D.E., Zhao F.J. (2020). Mutation in *OsCADT1* enhances cadmium tolerance and enriches selenium in rice grain. New Phytol..

[67] Kawahara Y., de la Bastide M., Hamilton J.P., Kanamori H., McCombie W.R., Ouyang S., Schwartz D.C., Tanaka T., Wu J., Zhou S. (2013). Improvement of the *Oryza sativa* Nipponbare reference genome using next generation sequence and optical map data. Rice.

[68] Kim D., Paggi J.M., Park C., Bennett C., Salzberg S.L. (2019). Graph-based genome alignment and genotyping with HISAT2 and HISAT-genotype. Nat. Biotechnol..

[69] Livak K.J., Schmittgen T.D. (2001). Analysis of relative gene expression data using real-time quantitative PCR and the 2(-Delta Delta C(T)) Method. Methods.

[70] Pertea M., Pertea G.M., Antonescu C.M., Chang T.-C., Mendell J.T., Salzberg S.L. (2015). StringTie enables improved reconstruction of a transcriptome from RNA-seq reads. Nat. Biotechnol..

[71] Sherman B.T., Hao M., Qiu J., Jiao X., Baseler M.W., Lane H.C., Imamichi T., Chang W. (2022). DAVID: A web server for functional enrichment analysis and functional annotation of gene lists (2021 update). Nucleic Acids Res..

[72] Kolde R. (2019). Pheatmap: Pretty Heatmaps.

