# Supplementary material for: Transcriptome-Wide Analysis of Core Transcription Factors Associated with Defense Responses in Autotetraploid versus Diploid Rice under Saline Stress and Recovery
Source: Int J Mol Sci. 2023 Nov 5;24(21):15982. doi: 10.3390/ijms242115982 (PMC10650042; doi:10.3390/ijms242115982)
Supplement: Supplementary file 1 [file ijms-24-15982-s001.zip › ijms-2631516-Figure S1-S3.pdf]

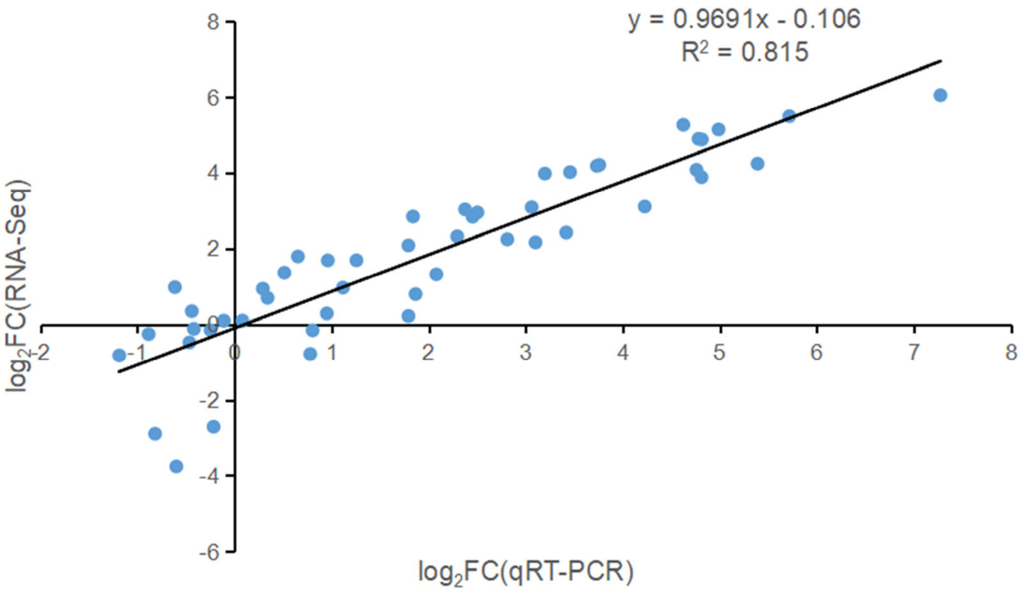

Figure S1: The quantitative real–time (qRT–PCR) expression profiles of seven selected TF genes in response to saline stress. The reference line represents the linear relationship between the fold change analysis of RNA–seq and qRT–PCR

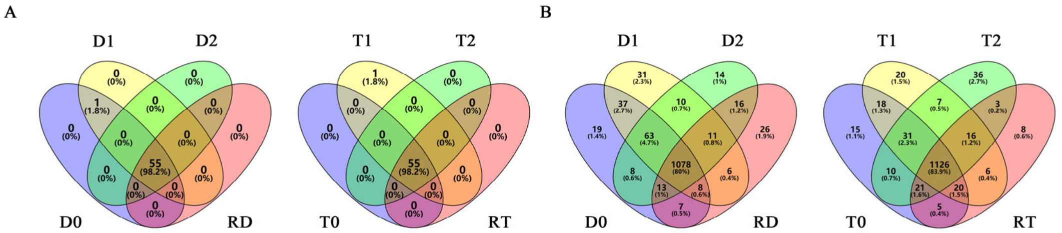

Figure S2: Venn diagrams representing common and specific (A) transcription factor family and (B) transcription factor genes in GFD–T and GFD–D at four different time points. D0/T0: diploid or tetraploid without NaCl stress; D1/T1: diploid or tetraploid seedlings were treated with NaCl for 3 h; D2/T2: diploid or tetraploid seedlings were treated with NaCl for 24 h; RD/RT: recovery culture of diploid or tetraploid seedlings for 24 h.

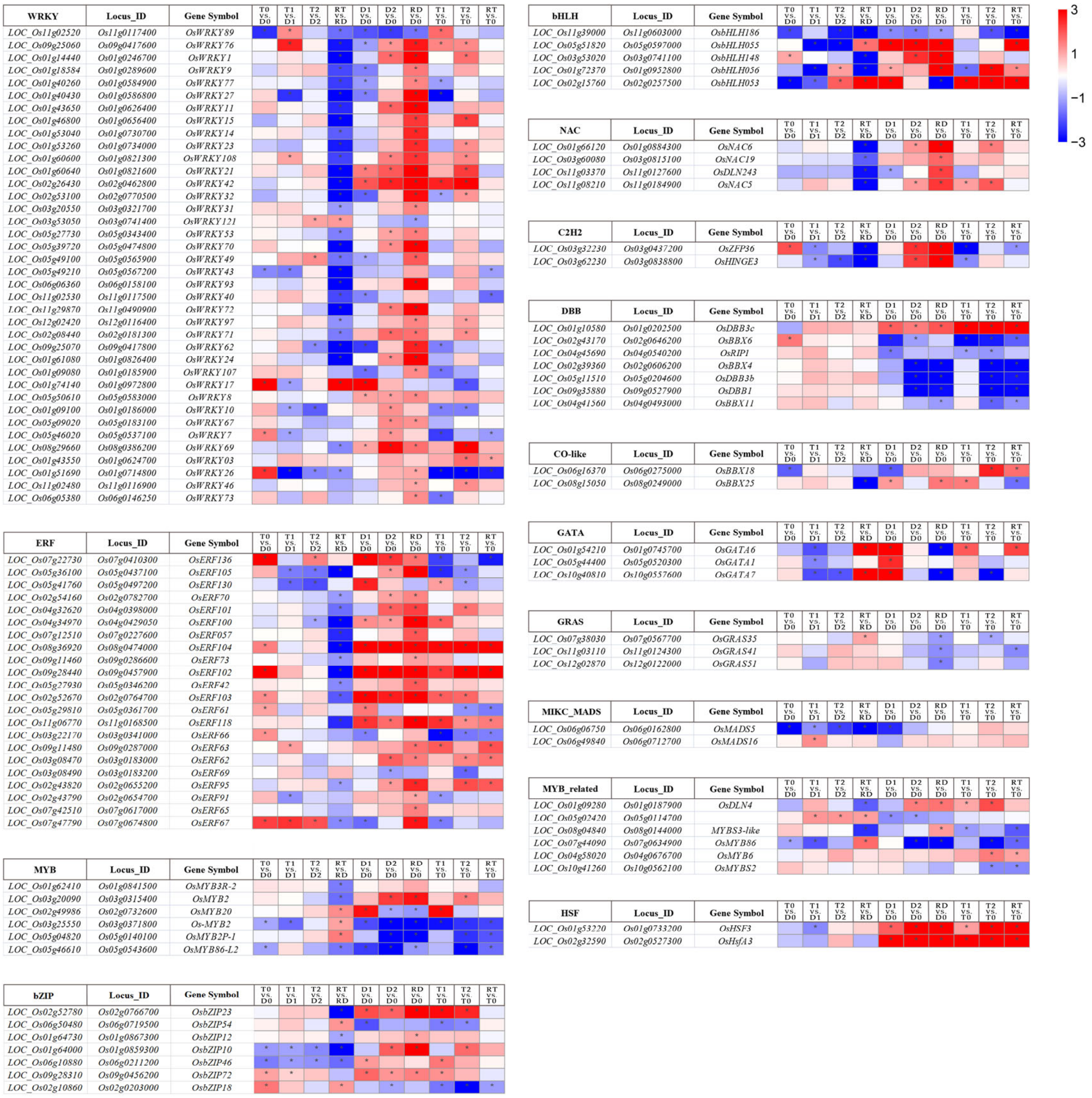

Figure S3: Heatmap analysis of core DE–TFs from fourteen TF families in intra–group and inter–group comparisons of GFD–T and GFD–D during saline stress and recovery period. Expression scores are shown as log2 fold change (FC) with * $p < 0.05$. D0/T0: diploid or tetraploid seedlings without NaCl stress; D1/T1: diploid or tetraploid seedlings treated with NaCl for 3 h; D2/T2: diploid or tetraploid seedlings treated with NaCl for 24 h; RD/RT: recovery culture of diploid or tetraploid seedlings for 24 h. DE-TFs: differentially expressed transcription factors.
